# A Systematic Review of the Costs Relating to Non-pharmaceutical Interventions Against Infectious Disease Outbreaks

**DOI:** 10.1007/s40258-021-00659-z

**Published:** 2021-06-11

**Authors:** Janetta E. Skarp, Laura E. Downey, Julius W. E. Ohrnberger, Lucia Cilloni, Alexandra B. Hogan, Abagael L. Sykes, Susannah S. Wang, Hiral Anil Shah, Mimi Xiao, Katharina Hauck

**Affiliations:** 1 Imperial College London, MRC Centre for Global Infectious Disease Analysis, Abdul Latif Jameel Institute for Disease and Emergency Analytics (J-IDEA), London, UK; 2 The George Institute for Global Health, School of Public Health, Imperial College London, London, UK

## Abstract

**Background:**

Non-pharmaceutical interventions (NPIs) are the cornerstone of infectious disease outbreak response in the absence of effective pharmaceutical interventions. Outbreak strategies often involve combinations of NPIs that may change according to disease prevalence and population response. Little is known with regard to how costly each NPI is to implement. This information is essential to inform policy decisions for outbreak response.

**Objective:**

To address this gap in existing literature, we conducted a systematic review on outbreak costings and simulation studies related to a number of NPI strategies, including isolating infected individuals, contact tracing and quarantine, and school closures.

**Methods:**

Our search covered the MEDLINE and EMBASE databases, studies published between 1990 and 24 March 2020 were included. We included studies containing cost data for our NPIs of interest in pandemic, epidemic, and outbreak response scenarios.

**Results:**

We identified 61 relevant studies. There was substantial heterogeneity in the cost components recorded for NPIs in outbreak costing studies. The direct costs of NPIs for which costing studies existed also ranged widely: isolating infected individuals per case: US$141.18 to US$1042.68 (2020 values), tracing and quarantine of contacts per contact: US$40.73 to US$93.59, social distancing: US$33.76 to US$167.92, personal protection and hygiene: US$0.15 to US$895.60.

**Conclusion:**

While there are gaps and heterogeneity in available cost data, the findings of this review and the collated cost database serve as an important resource for evidence-based decision-making for estimating costs pertaining to NPI implementation in future outbreak response policies.

**Supplementary Information:**

The online version contains supplementary material available at 10.1007/s40258-021-00659-z.

## Key Points for Decision Makers

**Table:** 

There are gaps in existing non-pharmaceutical intervention cost data literature both geographically and by intervention.
Publishing costs for the SARS-CoV-2 pandemic outbreak responses will help fill these gaps.

## Introduction

The SARS-CoV-2 pandemic has put unprecedented strain on health systems around the world and brought to the fore the importance of establishing effective infectious disease outbreak response strategies to protect population health. Countries have had to implement non-pharmaceutical interventions (NPIs) in the absence of suitable vaccines and other medical interventions as part of their outbreak mitigation or suppression strategies [1]. NPIs often come with a direct and socioeconomic cost, as in addition to administration costs or lost wages, they often require changes in behavioural patterns, which in turn, have wider impacts such as productivity losses or reduced consumption.

Considering that NPIs have been adopted at scale by nearly all countries globally as a response to SARS-CoV-2 in 2020, and for prolonged periods of time, discussion regarding the burden brought by the costs associated with NPIs has become commonplace [2]. Countries were making decisions on suppression and mitigation strategies early on in the pandemic while ignoring the costs associated with these interventions when implemented on a large scale. As the costs and scale of interruption associated with the SARS-CoV-2 pandemic and control interventions are becoming apparent, the current pandemic also acts as a prompt to consider the costs of NPIs associated with outbreak response strategies generally. Knowing the costs of NPIs would help countries to make informed evidence-based decisions when deciding on NPIs for future outbreaks, leading to more resilient health systems. This being said, NPI costs remain relevant for SARS-CoV-2 as although vaccines are being rolled out, it will likely still be many months before populations are vaccinated at a level that would allow for NPIs to be completely lifted around the globe.

Previous literature reviews on NPIs have focussed on particular pathogens or NPIs. Examples of such reviews include school closures for influenza pandemics, or travel bans, [3, 4]. To our knowledge, a comprehensive systematic review covering all the literature on costs for all settings and pathogens for community-based NPIs does not yet exist. There is a great need for this review, as we need to map what is known about the costs of these community-based NPIs for different settings and for different pathogens so that knowledge gaps can be identified and filled to improve the evidence available, and to inform future strategies relating to outbreak response in cases where pharmaceutical interventions are not available or feasible.

The aim of this review is to provide a comprehensive overview of the existing literature on the costs of community-based NPIs. We cover the costs of NPIs relating to isolating infected individuals, contact tracing and quarantine, travel and flight restrictions, social distancing, point-of-entry measures, and personal protection and hygiene in relation to outbreaks in non-hospital settings. We include studies that are both presenting outbreak response costs as well as simulation studies.

## Methods

The objective of this literature review was to capture the literature on costs of community-based NPIs for different types of outbreak settings. Studies of interest were separated into two categories: outbreak costing studies, and simulation studies. We define outbreak costing studies as studies which contain observed primary costs for components of NPI implementation in outbreak response scenarios, which could be used in economic models and future policy decisions. Simulation studies, on the other hand, are more useful for identifying relevant literature on applying different NPI modelling strategies, or for policy-making purposes where comparative costs between different strategies are considered.

### Inclusion and Exclusion Criteria

Table 1 presents the inclusion and exclusion criteria for our review. We considered outbreaks affecting the human population (excluding outbreaks in animals) in any location published from 1990 onwards for any non-chronic infectious disease. We only included original articles or reviews published or accepted in a peer-reviewed journal or published reports from official public health bodies, such as the Centers for Disease Control, published in English. We focused on interventions in the community, as these are most likely to provide useful information to inform response strategies for larger outbreaks, such as SARS-CoV-2. Studies involving hospital employees were included if the hospital was within a community outbreak (e.g., costs of home isolation of infected healthcare workers during community-wide H1N1 influenza outbreak), otherwise we excluded hospital-based studies as we deemed them to not be representative of a general community outbreak scenario. Studies for which pharmaceutical intervention costs could not be separated from non-pharmaceutical intervention costs were excluded.

**Table 1:** Inclusion and exclusion criteria for the literature review

Inclusion	Exclusion
Contains cost data of defined interventions^a^ of interest or on items relating to these interventions in pandemic, epidemic, or outbreak scenarios related to humans	Does not contain cost data on direct OR socio-economic costs of defined interventions^a^ in pandemic, epidemic, or outbreak scenarios
Original articles or reviews published or accepted in a peer-reviewed journal or reports	Intervention done to animals
Modelling studies estimating costs for defined interventions^a^	Cost data for diseases in endemic settings or chronic illnesses
	Duplicates
	Not in English
	Editorials, commentaries, letters, conference abstracts. (items that are not original articles or reviews published or accepted in a peer-reviewed journal or reports)

^a^Defined interventions: isolation of infected individuals, contact tracing and quarantine, travel and flight bans, social distancing, measures at point-of-entry, personal protection and hygiene, community stay at home orders

### Non-pharmaceutical Interventions of Interest

We considered NPIs that related to isolating infectious individuals or contacts, or included community interventions aiming to reduce community contacts through social distancing, such as curfews, school closures, workplace contact reductions (through closure, workplace or school absenteeism, or remote working), and wider crowd avoidance measures such as avoiding public transport and events. We also included stricter community-wide social distancing interventions, such as community-wide or country-wide stay-at-home orders. Additionally, we included travel restrictions and border closures and measures at points of entry, focussing on scans or screens done when individuals are entering or exiting a country or region. For personal protection measures, we included community-based usage of face masks, gloves, hand hygiene measures, and sanitisation protocols of contaminated surfaces. Table 2 presents a full list of NPIs considered.

**Table 2:** A list of non-pharmaceutical interventions considered in this literature review

Non-pharmaceutical intervention	Sub-categories of intervention
Isolation of infected individuals	Non-hospital case quarantine
Tracing and quarantine of contacts	Contact tracing Non-hospital contact quarantine Household quarantine
Social distancing	Curfew School closure Workplace closure Workplace absenteeism Working from home Crowd avoidance
Strict social distancing	Community stay-at-home orders Country stay at home orders
Travel & flight bans	Any sort of travel restriction, ban, or border closure
Measures for persons at point-of-entry	Scans/screens done when entering/exiting a country/region
Personal protection & hygiene	Face masks Hand hygiene (hand washing, sanitising, etc.) Sanitising contaminated surfaces Using gloves

### Intervention Costs of Interest

For outbreak costing studies, we extracted costs incurred by the individual affected by the NPI (e.g., wages lost due to home quarantine), costs incurred by the government, business, or public health body due to administering the NPI (e.g., contact tracing activities, face masks), and information relating to labour (e.g., number of hours spent on contact investigation per contact). We did not extract costs that were linked to pharmaceutical interventions that were combined with an NPI (e.g., vaccine administration costs) or case management in hospitals. For simulation studies, we included studies which presented costs separately from pharmaceutical costs. We covered simulation studies presenting any kind of financial impact, from cost calculations to reductions in gross domestic product.

For the quarantine of infectious individuals and their contacts, we considered cost or labour data relating to quarantine in a non-hospital setting. We excluded the costs of quarantine in hospital settings, as we considered them to not be representative of the costs relating to a community-based quarantine intervention due to the additional costs of components such as medical staff and hospital beds. We included costs relating to testing for infection only if testing was a component of the case identification and contact tracing protocol. With regard to contact tracing, we were interested in the community investigation costs and not pharmaceutical intervention costs. This meant that studies which did not separate non-pharmaceutical contact investigation costs from the vaccine or prophylactic treatment costs were excluded.

All costs from the outbreak-costing studies were converted to 2020 USD (mid-year, June) by first inflating the cost in its original reported currency to 2020 and then converting the value to USD [5]. The initial consumer price index was matched to the month when the intervention occurred, or the mid-point of the intervention timing if it lasted for a longer time-frame. The method of inflation adjustment followed the following formula:

$${\text{Initial}}\;{\text{value}} \times \frac{{{\text{Consumer}}\;{\text{Price}}\;{\text{Index}}\;2020}}{{{\text{Consumer}}\;{\text{Price}}\;{\text{Index}}\;{\text{initial}}}}.$$

The Consumer Price Index used was that of the International Monetary Fund [6]. Bloomberg’s currency conversion charts were used for currency conversion to USD [7].

The outputs of simulation studies were not converted as they are often the outcome of multiple inputs and assumptions, meaning that converting their outcomes would not be appropriate.

### Search Strategy

We searched the MEDLINE and EMBASE databases for studies pertaining to the NPIs described in Table 1 on 24 March 2020. The search strategy, including the search strings, can be found in the supplement file called “Search strategy”. The two databases were chosen, as they are the major databases that cover literature on pandemics, epidemics, and outbreaks, leading us to believe that other databases would have likely only added duplicate references.

The literature review was conducted systematically, meaning that at both title and abstract screening and full text screening, each paper was examined by two reviewers of the review team, these included ABH, ALS, HAS, JS, JWEO, LC, LD, MX, and SSW. Conflicts were resolved in conflict resolution meetings between two members of the review team (JS, JWEO). We followed a first-degree snowball approach for the relevant reviews identified in our screening process, where studies in the identified review were evaluated for inclusion, but second-degree references (references of references) were not. We enquired about full texts of difficult-to-find studies through the British Library.

We adapted the *British Medical Journal* guidelines for assessing economic studies [8]. Our quality assessment contained 26 points, some of which were exclusive only to simulation studies. We categorised studies as low, medium, or high quality based on the proportion of “Yes” scores to the total number of points that were applicable to the study. Studies of low quality covered 25 % or fewer of the points, studies of moderate quality covered between > 25 % and < 75 % of the points, while studies of high quality covered ≥ 75 % of the points. See supplementary spreadsheet for individual quality assessment scores for each study.

We registered the literature review on PROSPERO (review ID CRD42020177418).

## Results

### Studies Identified

**Fig. 1:**
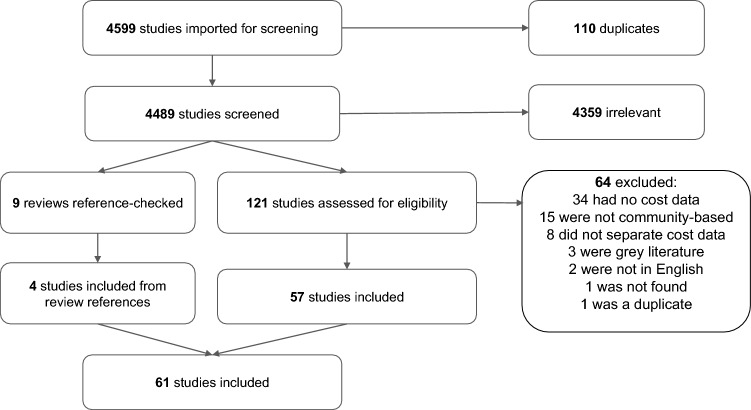
Flow diagram of literature review and studies identified, included, and excluded at each stage of the review process

We identified 4599 studies for title and abstract screening, 4359 of which were excluded and 121 studies were assessed for eligibility during full-text screening. Additionally, nine reviews were reference checked. Consequently, we identified a total of 61 relevant studies with cost information on relevant NPIs (27 costing studies and 34 simulation studies). Of these 61 studies, 4 were identified through reference-checking reviews relevant to the NPIs of interest, while the remaining 57 were identified directly through the MEDLINE and EMBASE search (see Fig. 1). At the full-text screening phase, there was disagreement between reviewers regarding inclusion for 27 (22.3%) studies. Of the included studies, 1.6% (1/61) were assessed as being of low quality, 44.2% (27/61) were assessed as being of moderate quality, and 54.1% (33/61) were assessed as being of high quality (see supplementary spreadsheet for full quality assessment for each study).

**Fig. 2:**
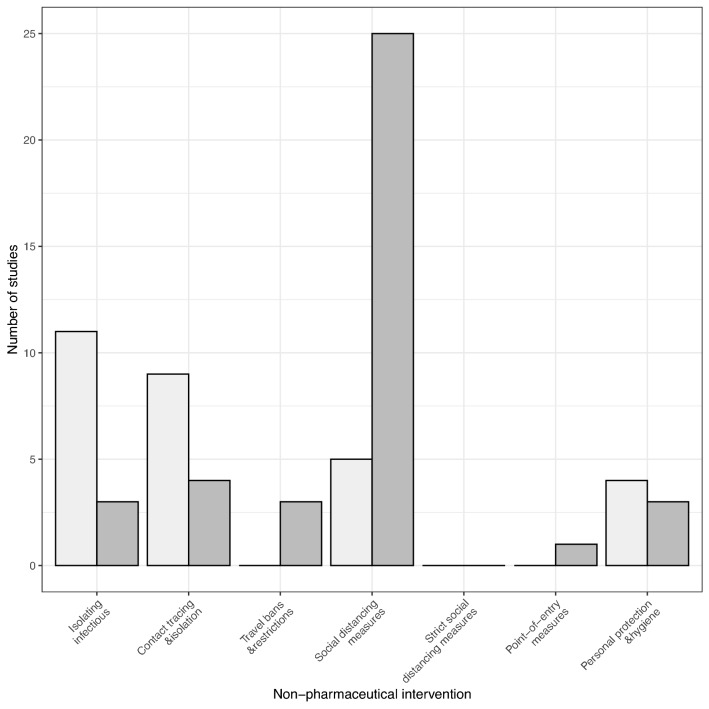
Bar plot of the number of studies that contain cost data for each non-pharmaceutical intervention for outbreak costing studies (light grey) and simulation studies (dark grey)

In the following sections, we present the identified cost and simulation evidence for each category of NPI (see Fig. 2 for number of studies by intervention). Due to the heterogeneity of costs recorded for the implementation strategies, it was deemed inappropriate to pool cost estimates. Hence, here we present the range of costs identified when there are comparable intervention components.

### Non-hospital Isolation of Infected Individuals

We identified 11 outbreak costing studies relating to isolating infected individuals at home or in a hotel in outbreak scenarios [9–19], and three simulation studies that explored the costs of isolating infected individuals [20–22]. Table 3 summarises the available cost information from these studies in 2020 USD (US$) converted to unit costs where applicable for the outbreak costing studies, and Table 4 summarises the simulation studies in the reported currencies (see supplementary spreadsheet for original extracted data in its original currencies and units). The available studies were focused largely in Europe, North America, and China, with few studies from low- and middle-income countries (LMICs). The pathogens were vaccine-preventable diseases (measles, pertussis), diarrhoeal pathogens (norovirus, *Escherichia coli*), or respiratory pathogens (H1N1 influenza, SARS).

**Table 3:** Identified outbreak costing studies that contained cost or labour information on non-pharmaceutical interventions

1st author, Publication year [Reference]	Year of intervention	Country	Pathogen	Target group	Intervention characteristic	Cost measured	Cost
**Isolating infectious individuals**							
Christie, 1995 [9]	1993	USA	Pertussis	Healthcare workers during pandemic influenza	Furloughing isolated infected individuals	Cost per case	971.26
					Case confirmation	Laboratory testing (per sample)	71.42
Wahl, 2011 [10]	2009	Norway	*Escherichia coli*	Parents of children in child-care	Isolating infected children	Work-days lost by parents per infected case	25.38
Ma, 2017 [11]	2015	China	Measles	Office workers	Isolating infected	Mean work-days lost	8.7 (95 % CI 8.5–8.9)
						Mean wages lost	593.14 (95 % CI: 546.03–640.24)
Galante, 2012 [12]	2009–2010	Spain	H1N1	Community	Isolating infected	Cost of work absenteeism	672.05
						Cost of work absenteeism due to caregiving responsibilities	57.51
Mota, 2011 [13]	2009	Brazil	H1N1	Physician in community outbreak	Isolating infected	Staff replacement (cost per day)	276.66
						Productivity loss (cost per day)	122.85
				Nurse in community outbreak	Isolating infected	Staff replacement (cost per day)	82.84
						Productivity loss (cost per day)	98.98
				Nurse assistant in community outbreak	Isolating infected	Staff replacement (cost per day)	53.85
						Productivity loss (cost per day)	50.65
Sugerman, 2010 [14]	2008–2009	USA	Measles	Children	Isolating infected children	Mean cost per case	946.57
					Case confirmation	Laboratory work (hours per confirmed case)	322
						Laboratory materials and work (cost per confirmed case)	641.35
Gallagher, 2013 [15]	2009	USA	*Escherichia coli*	Parents of isolating children	Isolating infected children	In-home childcare cost	1814.05
Ooi, 2005 [16]	2003	Singapore	SARS	Community	Quarantine enforcement and surveillance	Cost per case	340.23
					Quarantine command centre	Cost per case	71.63
					Quarantine allowance	Cost per case	322.32
					Emergency call centre and ambulance	Cost per case	71.63
Wang, 2012 [17]	2009	China	H1N1	Community	Isolation of infected	Inspection cost per case	29.48
						Disinfectant cost per case	20.95
						Home medical observation cost per case	90.75
Coleman, 2012 [18]	2010	USA	Measles	Community	Case confirmation	Labour hours (per sample)	0.5
						Labour costs (per sample)	17.86
						Screening kit cost (per sample)	141.9
					Quarantine of infected individuals	Labour hours per case at quarantine stations	4
						Labour costs per case at quarantine stations	330.28
Bownds, 2003 [19]	1998	USA	Hepatitis A	Community	Case quarantine	Cost of laboratory tests and procedures (per sample)	20.05
						Productivity loss due to staying at home when ill (per case)	4038.23
**Tracing and quarantine of contacts**							
Wang, 2012 [17]	2009	China	H1N1	Community	Contact quarantine	Quarantine at home (per person)	40.73
						Quarantine at hospital (per person)	724.94
						Quarantine in hotel (per person)	1062.32
					Contact observation	Isolated observation	4778.33
					Laboratory costs	Network laboratory	140.33
						Specimen collection	26.41
						Virus isolation and identification	237.65
						Nucleic acid detection	528.1
						Serology tests	66.01
Parker, 2006 [23]	2005	USA	Measles	Community	Contact tracing	Investigation hours	11.9
						Laboratory work hours	9.33
Pike, 2020 [24]	2016–2017	USA	Mumps	Community	Contact tracing for outbreak containment	Overall costs (total)	941104.38
						Labour costs (total)	503687.63
						Travel costs (total)	88927.80
						Personnel hours (total)	12585
					Laboratory costs	Tests (per sample)	18.53
						Supplies and equipment (total)	114861.53
Rosen, 2018 [25]	2013	USA	Measles	Community	Contact tracing activities	Community outreach (h per identified contact)	0.29
						Administration (h per identified contact)	0.13
						Advertising (h per identified contact)	8.63
					Laboratory	Laboratory personnel (h per sample)	57.63
						Laboratory supplies and testing ($ per sample)	214.96
Sugerman, 2010 [14]	2008	USA	Measles	Children	Contact tracing	Investigation (h per contact)	0.49
Dayan, 2005 [26]	2004	USA	Measles	Community	Contact tracing	Investigation (h per contact)	0.75
						Public information (cost per contact)	1.80
Flego, 2013 [27]	2011	Australia	Measles	Community	Contact tracing	Personnel cost (per contact)	23.96
						Personnel time (mean h per contact)	0.63
						Laboratory (cost per tested contact)	25.88
						Telephone (cost per contact call)	0.51
						Stationery and mail (cost per contacted contact)	2.8
Ma, 2017 [11]	2015	China	Measles		Contact tracing and surveillance	Cost (per contact)	42.99
						Time (h per contact)	2.12
					Field investigation	Cost of contact tracing and sample collection (per contact)	1.01
						Hours taken to contact trace (per contact)	0.07
					Laboratory testing of contacts	Cost of laboratory work (per sample)	11.83
						Hours of laboratory work (per sample)	101.91
						Cost of kit (per sample)	37.76
Gallagher, 2013 [15]	2009	USA	*Escherichia coli*	Children	Laboratory testing of contacts	Cost (per sample)	183.15
**Social distancing**							
Borse, 2011 [28]	2009	USA	H1N1	Parents of elementary school children	School closure	Households where at least 1 adult took time off work (%)	17
						Households where no adults took time off work (%)	83
Chen, 2011 [29]	2009	Taiwan	H1N1	Parents of elementary school children	School closure	Average income loss (per household)	33.76
Gift, 2010 [30]	2009	USA	H1N1	Parents of elementary school children	1 week school closure	% of households where 0 days of work were lost	78.5
						% of households where 1 days of work were lost	6.1
						% of households where 2 days of work were lost	3.3
						% of households where 3 days of work were lost	1.9
						% of households where 4 days of work were lost	1.9
						% of households where 5 days of work were lost	8.4
Johnson, 2008 [31]	2006	USA	Influenza B	Households with elementary school children	2-week school closure	Households where adults missed at least 1 day of work (%)	3.2
Russell, 2016 [32]	2013	USA	ILI	Households with school children	4 work-day school closure	Cost of childcare for households that required it (median, min-max)	111.95 (34.70–167.92)
**Personal protection and hygiene measures**							
Tracht, 2012 [33]	2009	USA	H1N1	Community	N95 mask	Cost per mask	2.14
Ma, 2017 [11]	2015	China	Measles	Office workers	Disposable mask	Cost per mask	0.32
					Hand sanitiser	Cost per bottle	5.09
Mukerji, 2017 [34]	2008–2010	China	Influenza	Healthcare workers during community transmission	Medical mask	Cost per mask	0.15
					N95 mask	Cost per mask	0.87
Baracco, 2015 [35]	2013	USA	Influenza	Healthcare workers during community transmission	N95 mask	Min/max cost per mask	0.28–0.73
					Reusable mask	Min/max cost per mask	27.99-55.97
					Set of filters for reusable mask	Cost per set	2.8
					Air-purifying device	Min/max cost per device	559.75–895.60
					Air-purifying device battery	Cost per battery	279.87
					Additional hood for purifier	Cost per hood	33.58
					Additional tubes for purifier	Cost per tube	33.58

All costs converted to 2020 USD unless indicated otherwise, original costs presented in supplementary spreadsheet

*AUD* Australian Dollars, *CAD* Canadian Dollars, *CGE* Computable General Equilibrium, *GDP* Gross Domestic Product, *h* hours, *ICER* incremental cost-effectiveness ratio, *R0* basic reproduction number, *SEIR* susceptible-exposed-infected-recovered, *SEIQR* susceptible-exposed-infected-quarantined-recovered, *SI* susceptible-infected, *SIR* susceptible-infected-recovered

**Table 4:** Identified simulation studies that contained cost or labour information on non-pharmaceutical interventions

1st author, Publication year [Reference]	Year of intervention	Country	Pathogen	Intervention type	Intervention characteristic	Cost measured	Cost	Model type
**Isolating infectious individuals**								
Agusto, 2013 [20]	NA	NA	Avian influenza	Isolation of infected	Total cost and ICER of isolating infectious individuals	Avian strain (total cost in theoretical units [ICER])	89648 (0.18411)	Deterministic compartmental SI transmission model + incremental cost-effectiveness ratio
						Mutant strain (total cost in theoretical units [ICER])	71133 (−0.08633)	
						Both strains (total cost in theoretical units [ICER])	16441 (−0.68322)	
Yarmand, 2010 [21]	NA	USA	H1N1	Isolation of infected	Cost effectiveness of isolating infectious individuals vs vaccination	Compares various percentages of isolation against a vaccination policy in a theoretical manner—no single cost reported		compartmental SEIR transmission model + linear function of costs, optimising cost-effectiveness of a response consisting of isolation and vaccination
Mubayi, 2010 [22]	NA	Hong Kong	SARS	Isolation of infected	Cost effectiveness of case isolation for a contact tracing strategy that has a per-capita rate independent of number infected	Presents a variation of costs for multiple contact-tracing parameter values		Compartmental SEIQR transmission model + linear cost function to model cost-effectiveness, incremental cost-effectiveness also evaluated
					Cost effectiveness of case isolation for a contact tracing strategy that has a per-capita rate that is proportional to number infected	Presents a variation of costs for multiple contact-tracing parameter values		
					Cost effectiveness of case isolation for a contact tracing strategy that has a per-capita rate that is finite and saturates	Presents a variation of costs for multiple contact-tracing parameter values		
**Tracing and quarantine of contacts**								
Li, 2013 [36]	2009	China	H1N1	1-week contact quarantine for a 60-day intervention period	Contact tracing and contact quarantine in hotel	Total cost (USD 2009)	2560000	deterministic compartmental SEIR transmission model + cost-effectiveness with a counterfactual of no contact quarantine
Orset, 2018 [37]	NA	France	Pandemic influenza	Contact quarantine	Contact quarantine at home	Presents a variety of hypothetical thresholds and compliance levels		Cost-benefit analysis and probit model
Gupta, 2005 [38]	NA	Canada	SARS	Contact quarantine	Total cost of quarantine	Total cost of primary wave (million CAD 2003)	12.2	simple population level transmission model + analysis of cost savings with a counterfactual of no contact quarantine
						Total cost of secondary wave (million CAD 2003)	13	
						Total cost of tertiary wave (million CAD 2003)	17	
					Total savings due to quarantine	Total saving in primary wave (million CAD 2003)	279	
						Total savings in secondary wave (million CAD 2003)	274	
						Total savings in tertiary wave (million CAD 2003)	232	
Mubayi, 2010 [22]	NA	Hong Kong	SARS	Contact quarantine	Cost effectiveness of contact quarantine per-capita rate independent of number infected	Presents a variation of costs for multiple contact-tracing parameter values		Compartmental SEIQR transmission model + linear cost function to model cost-effectiveness, incremental cost-effectiveness also evaluated
					Cost effectiveness of contact quarantine per-capita rate that is proportional to number infected	Presents a variation of costs for multiple contact-tracing parameter values		
					Cost effectiveness of contact quarantine per-capita rate that is finite and saturates	Presents a variation of costs for multiple contact-tracing parameter values		
**Travel and flight bans and restrictions**								
Epstein, 2007 [39]	NA	USA	Pandemic influenza	Air travel restrictions	Cost of air travel restrictions	Cost of shutting down major airlines (billion USD per annum)	93–100	Network-based individual-based SEIR transmission model + costs associated with epidemic and intervention along with benefits, counterfactual: no travel restriction
						Cost of shutting down major airlines (% GNP)	0.8	
						Labour cost of shutting down major airlines (billion USD per annum)	6	
Prager, 2017 [40]	NA	USA	Seasonal influenza	Travel restrictions	Travel restrictions' direct impact on GDP	Inbound international travel (% change in GDP)	−2.425	CGE model
						Outbound international travel ( % change in GDP)	−2.425	
						Domestic travel (% change in GDP)	−0.063	
			Pandemic influenza	Travel restrictions	Travel restrictions' direct impact on GDP	Inbound international travel (% change in GDP)	−19.833	
						Outbound international travel (% change in GDP)	−19.833	
						Domestic travel (% change in GDP)	−3.125	
Boyd, 2017 [41]	NA	New Zealand	Influenza	Border closure	Net costs and social benefits of border closure	Costs and benefits for multiple scenarios given for 12 week closer, 26-week closure, failed border closure		Transmission model + net costs and net societal benefits society calculated, counterfactual: cost of pandemic with no border closure
**Social distancing**								
Andradottir, 2011 [42]	NA	Canada	Pandemic influenza	School closure	5-day school closure and social distancing with 20 % contact limitation	Total cost (million CAD 2008)	125	Individual-based compartmental transmission model + cost calculation for interventions
Araz, 2012 [43]	NA	USA	Pandemic influenza	School closure	School closure for low transmission and low severity	12-week school closure cost (USD)	2,560,372,2 19	SEIR transmission model + cost calculation for interventions
						24-week school closure cost (USD)	5,120,744,439	
					School closure for high transmission and high severity	12-week school closure cost (USD)	2,560,372,219	
						24-week school closure cost (USD)	5,120,744,439	
Brown, 2011 [44]	2009	USA	H1N1	School closure	1-, 4-, and 8-week school closure	Present costs for school closure alone, the cost of school closure combined with costs of disease, and net costs of school closure (accounting for averted cases) for pandemics with an R0 of 1.2, 1.6 and 2.0		Agent-based transmission model + Monte Carlo cost-benefit simulation model
Halder, 2011 [45]	NA	Australia	H1N1	School closure	2-week school closure	Total cost (million USD 2010 per 100,000 population)	5.9	Individual-based transmission model + cost analysis
					4-week school closure	Total cost (million USD 2010 per 100,000 population)	6.6	
					8-week school closure	Total cost (million USD 2010 per 100,000 population)	11.6	
					Continuous school closure	Total cost (million 2010 USD per 100,000 population)	34.1	
					2-week school closure + 4-week 50 % workplace closure + 50 % community contact reduction	Total cost (million USD 2010 per 100,000 population)	21	
					2-week school closure + 4-week 50 % workplace closure	Total cost (million USD 2010 per 100,000 population)	21.1	
					2-week school closure + 50 % community contact reduction	Total cost (million USD 2010 per 100,000 population)	5.7	
					2-week school closure + 2-week 50 % workplace closure	Total cost (million USD 2010 per 100,000 population)	13.6	
					Continuous school closure + continuous 50 % workplace closure	Total cost (million USD 2010 per 100,000 population)	103	
Jones, 2013 [46]	NA	NA	Influenza	Social distancing	Contact reduction through social distancing	Presents costs for both transmission models and cost functions		Investigates two transmission models and linear and exponential increases in intervention costs when optimising non-pharmaceutical interventions
Kelso, 2013 [47]	NA	Australia	Influenza A	Social distancing	School closure, 50 % workplace reduction, 50 % community contact reduction	Presents intervention costs for five pandemic severities, with varying combinations of the three social distancing measures		SEIR compartmental model + cost analysis including direct healthcare costs and productivity loss
Keogh-Brown, 2010 [48]	NA	UK, France, Belgium, Netherlands	Influenza	Social distancing measures	School closure	GDP loss due to school closure (% GDP loss min-max)	1.32–3.20	unspecified transmission model and one-country CGE model
					Prophylactic absenteeism	GDP loss due to prophylactic absenteeism (% GDP min-max)	0.94–2.34	
Lempel, 2009 [49]	NA	USA	Pandemic influenza	Worker absenteeism due to school closure	Length of school closure	2 weeks (base cost (low-high cost) in billion USD 2008)	21.3 (5.2–23.6)	Economic cost calculation based on weekly earnings of caretakers multiplied by school closure length, no transmission model
						4 weeks (base cost (low-high cost) in billion USD 2008)	42.6 (10.6–47.1)	
						6 weeks (base cost (low-high cost) in billion USD 2008)	63.9 (15.6–70.7)	
						12 weeks (base cost (low-high cost) in billion USD 2008)	127.8 (31.3–141.3)	
						Weekly cost per student (base cost (low-high cost) in billion USD 2008)	142 (35–157)	
Maharaj, 2012 [50]	NA	NA		Social distancing	Reduction of contacts	Presented for a range of infectiousness levels and show its effect on the net economic benefit of social distancing		Compartmental SIR transmission model with and without small-world interactions with calculation of net economic benefit
Milne, 2013 [51]	NA	Australia	Pandemic influenza	School closure, workplace closure, community contact reduction	Continuous school closure + continuous workplace closure	Total cost per member of population (AUD, low severity-high severity)	1217–4804	Individual-based transmission model + costing model to determine economic cost to society
					Continuous school closure + 4-week 50 % community contact reduction	Total cost per member of population (AUD, low severity-high severity)	519–3826	
					Continuous school closure + 4-week workplace closure and 50 % community contact reduction	Total cost per member of population (AUD, low severity-high severity)	654–3882	
					Continuous school closure + continuous 50 % community contact reduction	Total cost per member of population (AUD, low severity-high severity)	447–2275	
					Continuous school closure, workplace closure, and 50 % community contact reduction	Total cost per member of population (AUD, low severity-high severity)	1116–2603	
Morin, 2014 [52]	NA	NA		Community contact reduction	Varied reduction percentages	Presents interface between numbers infected and susceptible where community contact reduction is considered worth the cost for each transmission model		Three transmission models: SI, SIR, SEIR and costs to society
Nishiura, 2014 [53]	NA	Japan	Pandemic influenza	School closure	Varying lengths of school closure (0–50 days)	ICER presented for varying lengths of school closure and varying infectiousness		Renewal process transmission model + incremental cost effectiveness ratio
Perlroth, 2010 [54]	NA	USA	Pandemic influenza	Social distancing	Adult and child social distancing + school closure	Total cost per person in a setting with R0 of 2.1 and case fatality rate 1 %) (USD 2009)	1400	Agent-based network model of transmission with calculation of costs
						Total cost per person in a setting with R0 of 1.6 and case fatality rate 0.25 % (USD 2009)	1370	
					Adult and child social distancing	Total cost per person in a setting wit R0 of 2.1 and case fatality rate 1 % (USD 2009)	490	
						Total cost per person in a setting with R0 of 1.6 and case fatality rate 0.25 % (USD 2009)	290	
					Quarantine	Total cost per person in a setting with R0 of 2.1 and case fatality rate 1 % (USD 2009)	720	
						Total cost per person in a setting with R0 of 1.6 and case fatality rate 0.25 % (USD 2009)	510	
					School closure	Total cost per person in a setting with R0 of 2.1 and case fatality rate 1 % (USD 2009)	1330	
						Total cost per person in a setting with R0 of 1.6 and case fatality rate 0.25 % (USD 2009)	1510	
Prager, 2017 [40]	NA	USA	Pandemic influenza	Social distancing	Social distancing measures’ impact on GDP	Public transportation (% impact on GDP)	−3.125	CGE model
						Workplace absenteeism (% impact on GDP)	−0.125	
						Parents keeping children from school (school avoidance + workplace absenteeism) (% impact on GDP)	− 0.012	
						Reduction in school attendance (% impact on GDP)	− 0.167	
			Seasonal influenza	Social distancing	Social distancing measures’ impact on GDP	Public transportation (% impact on GDP)	−0.063	
						Workplace absenteeism (% impact on GDP)	−0.038	
						Parents keeping children from school (school avoidance + workplace absenteeism) (% impact on GDP)	−0.006	
						Reduction in school attendance (% impact on GDP)	−0.083	
Reluga, 2010 [55]	NA	NA	NA	Social distancing	Contact reduction	Presents total costs and savings for varying social distancing efficiencies		SIR transmission model + cost calculation
Sadique, 2008 [56]	NA	UK	Pandemic influenza	School closure	2- 12-week school closure	Presents a range of school closure policies' effects on GDP with different labour impact assumptions		No transmission model, lost income calculated with human capital method
Sander, 2009 [57]	NA	USA	Pandemic influenza	School closure	26-week closure	Total cost per 1000 population (million USD)	2.72	Discrete time stochastic transmission model + cost calculation
Saunders-Hastings, 2017 [58]	NA	Canada	Pandemic influenza	Social distancing + personal hygiene	Community contact reduction + personal protective measures + voluntary isolation	Cost per life-year saved compared to no intervention (CAD)	6671	Discrete time population-level stochastic transmission model + cost calculation
					School closure + community contact reduction + personal protective measures + voluntary isolation and quarantine	Cost per life-year saved compared to no intervention (CAD)	260472	
Smith, 2013 [59]	NA	Thailand, South Africa, Uganda	pandemic influenza	School closure	1-week school closure	Presents cost per capita (in USD) of closure for 9 disease severity scenarios and % impact on GDP for each country for each scenario		CGE model
Smith, 2011 [60]	NA	UK	Pandemic influenza	Social distancing	School closure	Presents % impact on GDP for three disease severities		CGE model
					Prophylactic absenteeism	Presents % impact on GDP for three disease severities		
Smith, 2009 [61]	NA	UK	Pandemic influenza	School closure	School closure	Presents % impact on GDP for 3 case fatality rate scenarios and 3 clinical attack rate scenarios		CGE model
Wang. 2008 [62]	NA	NA		Community contact reduction	Closure of public spaces	Presents a theoretical interface of closure policy cost optimisation for outbreak scenarios		Scale-free SIR transmission model + cost calculation
Wong, 2016 [63]	NA	Hong Kong	H1N1	School closure	1- to 16-week closures and 3 different closure modes	Presents mean cost incurred for each closure scenario		SEIR compartmental transmission model + cost calculation
Xue, 2012 [64]	NA	Norway	Pandemic influenza	School closure	Various lengths of school closure	Presents costs and productivity losses for multiple lengths of school closure for 3 reproduction numbers		SEIR compartmental transmission model + cost-benefit calculation
Yaesoubi, 2016 [65]	NA	NA		School closure	Various lengths of school closure	Presents an interface of costs due to school closure		mathematical decision model with transmission dynamics (including SIR compartmental type structure) + cost optimisation
**Measures for persons at point-of-entry**								
Jacobson, 2016 [66]	NA	USA	Ebola	Point-of-entry screening	Screening cost per passenger	Costs for three different monitoring levels under two different policies (CDC and an alternative policy) are presented		Linear cost function applied to different scenarios
**Personal protection and hygiene measures**								
Jones, 2013 [46]	NA	NA	Influenza	Hygiene	Hygiene measures	Presents costs for both transmission models and cost functions		Investigates two transmission models and linear and exponential increases in intervention costs when optimising non-pharmaceutical interventions
Sardar, 2013 [67]	2008–2011	Zimbabwe	Cholera	Hygiene	Hand hygiene	Presents the optimal cost for 9 Zimbabwean locations for hygiene measures		Compartmental transmission model + cost function
Tracht, 2012 [33]	2009	USA	H1N1	Face masks	N95 face masks (10 %, 25 %, and 50 % usage)	Show savings gained by percentage of population who are using masks by age group		Compartmental SEIR transmission model + cost-benefit analysis

Costs are presented in their original currencies. The years of intervention and the country modelled are indicated where possible, but when no particular year or location is mentioned, they are specified as not applicable (NA)

*AUD* Australian Dollars, *CAD* Canadian Dollars, *CGE* Computable General Equilibrium, *GDP* Gross Domestic Product, *h* hours, *ICER* incremental cost-effectiveness ratio, *R0* basic reproduction number, *SEIR* susceptible-exposed-infected-recovered, *SEIQR* susceptible-exposed-infected-quarantined-recovered, *SI* susceptible-infected, *SIR* susceptible-infected-recovered

The costs covered by the 11 studies were highly heterogeneous, and included case confirmation costs, wages and productivity lost due to being in quarantine, costs of taking care of quarantined children at home. One study considered the costs incurred to the government due to isolating infected individuals during the SARS pandemic response in Singapore, and reported the costs of quarantine enforcement (US$340.23 [2020 values] per case), quarantine command centres (US$71.63 per case), quarantine allowance (US$322.32 per case), and emergency call centres (US$71.63 per case) [16]. There was one cost component, laboratory costs relating to case confirmation, that was covered by multiple studies. The ranges of laboratory costs are presented in section 3.8. The three simulation studies presented heterogeneous cost-related outputs, including the total cost of isolating infectious individuals, cost effectiveness of an isolation intervention versus vaccination, and cost effectiveness of isolating infectious individuals given different levels of contact tracing.

### Tracing and Quarantine of Contacts

We identified nine cost studies [11, 14, 15, 17, 23–27], and four simulation studies relating to contact tracing and contact quarantine in outbreak scenarios [22, 36–38]. Tables 3 and 4 summarise the cost information from these studies (the original extracted data in original currencies and units can be found in the supplementary spreadsheet). The studies were focussed on respiratory diseases (SARS and influenza) and vaccine-preventable diseases (measles and mumps). Much the same as isolation of infected individuals, the identified contact tracing papers were from North America and China.

As with case isolation, there was substantial heterogeneity in the types of costs recorded by the outbreak costing studies. Ranges of costs relating to laboratory testing are presented in section 3.8. The average hours spent on contact tracing was reported by five studies on measles outbreaks, and ranged from 0.5 to 11.9 hours [11, 14, 23, 26, 27]. The four simulation studies presented costs of contact tracing and quarantine at home and in a hotel.

### Travel and Flight Bans

We did not identify any outbreak costing studies on travel and flight bans or restrictions. However, we did identify three simulation studies [39–41], see Table 4 for further details and original extracted costs in the supplementary spreadsheet. All studies were on influenza, two were located in the USA and one in New Zealand. The two USA studies simulated the costs and GDP impacts of air travel restrictions, while the New Zealand study covered the full border closure.

### Social Distancing

We identified five costing [28–32] and 25 simulation studies on social distancing measures [40, 42–65], see Tables 3 and 4, respectively. Again, studies largely focussed on North America and Europe. All studies on a specified disease were on respiratory infections (various strains of influenza).

All costing studies reported only on school closures, and presented heterogenous costs, including days of work lost by parents, income loss due to lost work, and cost of childcare due to school closure. The simulation studies largely focussed on school closures and workplace absenteeism or closure, with many studies also considering combinations of community contact-reducing interventions.

### Measures for Persons at Point-of-entry

We identified one simulation study on NPI measures at point-of-entry [66]. This USA-based study simulated the costs per airline passenger of point-of-entry screening for Ebola for three different monitoring levels (Table 4).

### Personal Protection and Hygiene

While personal protection and hygiene measures in hospital settings for hospital-based outbreaks and nosocomial transmission were well documented, studies involving community-based outbreaks or community usage were rarer (see Tables 3 and 4 for costing and simulation studies, respectively). We identified four costing [11, 33–35] and three simulation studies on personal protection and hygiene measures [33, 46, 67]. The countries covered were USA, China and Zimbabwe. Most studies were on influenza, with one on measles and another on cholera. Face masks and hand sanitiser were the most covered interventions.

Three costing studies reported the costs of N95 face masks, which ranged from US$0.28 to US$2.14 [33–35]. Three simulation studies covered the savings due to different N95 face mask usage levels, and costs of general hygiene and hand hygiene measures.

### Laboratory Testing in Conjunction with Non-pharmaceutical Interventions

We included only studies where laboratory testing was combined with another NPI. We identified 11 costing studies that involved laboratory cost data, 4 of which were related to isolation of infectious cases [9, 14, 18, 19] and 7 were related to contact tracing (Table 3) [11, 15, 17, 23–25, 27]. We also identified one simulation study on laboratory testing in conjunction with an NPI, which was a cost-benefit analysis of an *E. coli* surveillance system in Colorado, USA (Table 4) [68].

The diseases covered were vaccine-preventable (measles, mumps, pertussis, hepatitis A), respiratory (H1N1), and *E. coli*. The only pathogen for which there were costs reported for more than one study was measles, where six studies contained information [11, 14, 18, 23, 25, 27]. For the measles studies, the reported costs of testing ranged from US$25.88 to US$641.00 per sample and data on hours ranged from 0.5 to 101.9 hours per sample. The reporting of components included in laboratory cost calculations were not consistent, as some studies reported cost of labour as part of laboratory costs and others did not.

## Discussion

In this study, we have reviewed the existing published literature on the NPIs of interest, covering both outbreak costing studies, which contain primary costs relating to NPIs in outbreak response, and simulation studies, which estimate costs of NPIs in outbreak response. Cost data are essential components of any evidence-based policy process and provide valuable information to be used alongside evidence of effectiveness to inform analyses pertaining to projected or actual estimates of the cost effectiveness and budget impact of implementation of different NPI strategies. There is variability in the levels of representation amongst the different NPI categories. Case isolation, contact tracing measures, and social distancing measures (in particular school closures) were well represented while travel restrictions, point-of-entry measures, and personal hygiene measures were less represented. Wider and stricter social distancing measures, such as community-wide measures, had not been covered in published literature before March 2020. Labour costs were often the most expensive component of isolating infected individuals and contact tracing, while laboratory costs also contributed greatly to the overall cost. There were nine papers that included NPIs and their costs, but did not present these costs separately from pharmaceutical (often vaccines and/or antivirals) interventions, and as such were excluded as the costs of the two different types of intervention could not be separated.

While we identified multiple costing studies that contained cost information for NPIs, providing meaningful and comparable summary statistics for them is difficult, as studies covered multiple locations and recorded different cost components relating to the community-based NPIs. Having a database of available cost information from outbreak costing studies is nonetheless useful for ease of locating relevant studies and cost components in future applications, such as for model parameterisation or in scenarios where policy-makers must compare the costs of different potential interventions. Studies covering the costs of travel bans and measures at point-of-entry would be a valuable addition to the existing literature. As many countries closed their borders or restricted entry into the country in the first months of 2020, this knowledge gap in cost data may be covered to an extent in literature that has been published since then [69]. The simulation studies also provided a range of model outputs, ranging from the total cost of implementing an NPI to the estimated impact on a country’s GDP. The database of simulation studies can act as a starting point for estimating the costs of a community-based NPI during an outbreak.

Published literature on the costs of NPIs for outbreaks in low-income settings was sparse. The majority of the studies identified were focused on North America, Europe, or Australia and New Zealand. While this may, in part, be by the exclusion of non-English studies and grey literature, this alone is likely not the only reason for the trend. In order to make well-informed pandemic response decisions, it is important that costings studies focus on low-income settings. The ongoing SARS-CoV-2 pandemic offers an opportunity for countries to collect outbreak response cost data for low-income settings to help fill this knowledge gap. We found that many studies were excluded from this review because they did not disentangle NPI costs from pharmaceutical intervention costs. It would be helpful if studies would present these costs separately to provide a clearer view of how each intervention contributes to the total cost of outbreak response.

The results published in this study are limited by the scope and extent of the literature review. This review covered literature that had been published by 24 March 2020. This necessarily limits the identification of publications to only those published up to the very beginnings of the SARS-CoV-2 pandemic. We did not identify any studies that recorded costs or simulated costs of strict social distancing measures (i.e., community stay-at-home orders) that are now commonplace across the globe for controlling the SARS-CoV-2 pandemic, due to this early cut-off point, which is a limitation of this study. This study does provide a broad review of the available epidemic- and pandemic-related research until COVID-19, and future research relating to COVID-19 outbreak costing and simulation studies can build on it. Extensive future research is indeed warranted to capture the cost of implementing NPIs, including strict social distancing, in relation to this unprecedented and devastating outbreak [70–72]. This review only covered studies from the MEDLINE and EMBASE databases, which publish studies on outbreaks. Studies that might have been exclusively available in the grey literature would not have been identified in this study.

This review presents the existing literature pertaining to the direct costs of implementing NPIs. There are important additional socioeconomic costs associated with the implementation of NPIs, such as the cost of businesses closing due to the intervention or the effects the NPIs have on mental health, the literature for which has not been covered by this review. Additionally, this review does not comprehensively summarise the cost effectiveness of all possible NPIs in outbreak response. Furthermore, as this review is focussed on the costs of public health measures, the costs of policies such as stimulus packages are beyond the scope of this review. The results of this study can be used for information purposes to provide a narrative summary of the cost of implementing historical NPI strategies, and to inform conversations around future planning for implementation of NPIs for pandemic response. The results of this study are also highly useful to inform future research, where numerous gaps or incomplete data were identified.

During the SARS-CoV-2 pandemic, community-based NPIs such as community-wide social distancing measures have been applied rapidly in countries across the globe, with little evidence available for estimating the costs of such an intervention *a priori*. Having easily accessible collated cost information on community-based NPI strategies will provide a valuable resource for informing future outbreak response policies, where cost data represent a vital component of any cost-effectiveness assessment of NPI options under consideration for implementation. Literature in this field will likely continue to accrue rapidly over the following months. Additional care should also be taken to collect and publish costs for low-income settings for future planning of pandemic financing. Maintaining a database summarising published literature on NPI costs in relation to outbreak response could be valuable for model parameterisation and outbreak response planning purposes.

## Supplementary Information

Below is the link to the electronic supplementary material.

**Supplementary document:** Literature review search strategy (PDF 40 kb)

**Supplementary spreadsheet:** Full information on costs presented in review papers and study quality (XLSX 323 kb)

## Declarations

### Availability of data and material

Data collected for this literature review is included in the Supplementary spreadsheet that accompanies this manuscript.
