# Supplementary material for: A Systematic Review of the Costs Relating to Non-pharmaceutical Interventions Against Infectious Disease Outbreaks
Source: Appl Health Econ Health Policy. 2021 Jun 11;19(5):673–97. doi: 10.1007/s40258-021-00659-z (PMC8192223; doi:10.1007/s40258-021-00659-z)
Supplement: Supplementary file 1 — Supplementary document: Literature review search strategy (PDF 40 kb) [file 40258_2021_659_MOESM1_ESM.pdf]

**Article title**	A systematic review of the costs relating to non-pharmaceutical interventions against infectious disease outbreaks
**Journal name**	Applied Health Economics and Health Policy
**Authors**	Janetta Skarp, Laura Downey, Julius Ohnberger, Lucia Cilloni, Alexandra Hogan, Abagael Sykes, Susannah Wang, Hiral Shah, Mimi Xiao, Katharina Hauck
**Affiliation**	Imperial College London, MRC Centre for Global Infectious Disease Analysis, Abdul Latif Jameel Institute for Disease and Emergency Analytics (J-IDEA), London, United Kingdom
**Corresponding author**	janetta.skarp13@imperial.ac.uk

Supplementary information: Search strategy

Search strings

(((isolation OR isolating OR confin* OR quarantin*) AND (infection* OR infected OR infectious OR ill OR diseased))
OR
(contact tracing AND contact*)
OR
((travel OR cruise OR flight OR train OR bus OR coach) AND (ban* OR restrict* OR limit*))
OR
(curfew* OR distancing OR ((gather* OR mass OR group) AND (ban OR restrict OR curb*)) OR ((school OR creche OR daycare OR kindergarten OR pre-school OR nursery) AND (closing OR closure OR close)) OR ((work* OR office OR firm OR factory) AND (non-attendance OR absenteeism OR closing OR closure OR close)) OR work* from home)
OR
((screen* OR scan*) AND (point of entry OR point-of-entry OR entry OR exit))
OR
((face mask OR facemask OR n95 OR n99) OR (glove OR gloves) OR (saniti?er) OR (disinfect* AND contaminat* AND (surface* OR door handle OR door knob OR area)) OR (hand-washing OR washing hands OR hand hygiene))
OR
(lockdown* OR (quarantin* AND (population OR community OR country OR nation OR state) AND (entire OR whole OR blanket OR everyone))))
AND
(cost* OR pric* OR econom* OR financ* OR ((econom* OR soci* OR financ*) AND (los* OR impact* OR effect* OR cost* OR consequence* OR burden*)))
AND
(outbreak* OR epidemic* or pandemic*)

Databases

- Medline
- EMBASE

Restrictions

- Published on or after 1990
- Search term is in title, abstract or keyword
